# Trigeminal Amyloidoma: Case Report and Review of Literature

**DOI:** 10.7759/cureus.3795

**Published:** 2018-12-28

**Authors:** Hamza Hashmi, Jugraj Dhanoa, Suresh Manapuram

**Affiliations:** 1 Oncology, University of Louisville School of Medicine, Louisville, USA; 2 Internal Medicine, University of Louisville School of Medicine, Louisville, USA; 3 Internal Medicine, Saint Francis Hospital and Medical Center, Grand Island, USA

**Keywords:** trigeminal neuraglia, amyloidoma, amyloidosis

## Abstract

Amyloid is an abnormal insoluble protein that can deposit in extracellular space. It can involve nearly any organ system and may manifest as a systemic process or focal lesion (amyloidoma). We present a rare case of localized amyloidosis with trigeminal nerve being the only site of involvement and no evidence of systemic disease. We also review literature relevant to trigeminal amyloidoma and make recommendations for diagnosis and treatment.

## Introduction

Amyloidosis is a generic term that refers to the extracellular tissue deposition of fibrils composed of low molecular weight subunits of a variety of proteins, many of which circulate as constituents of plasma [1]. These subunit proteins are derived, in turn, from soluble precursors which undergo conformational changes that lead to the adoption of a predominantly antiparallel beta-pleated sheet configuration. The two major forms of amyloidosis are the AL (primary) and AA (secondary) types. Other rare forms include dialysis-related amyloidosis, heritable amyloidosis, age-related systemic amyloidosis and organ-specific amyloid [1]. Clinical manifestations vary depending upon the type of amyloid and the distribution of deposition.

## Case presentation

**Figure 1:**
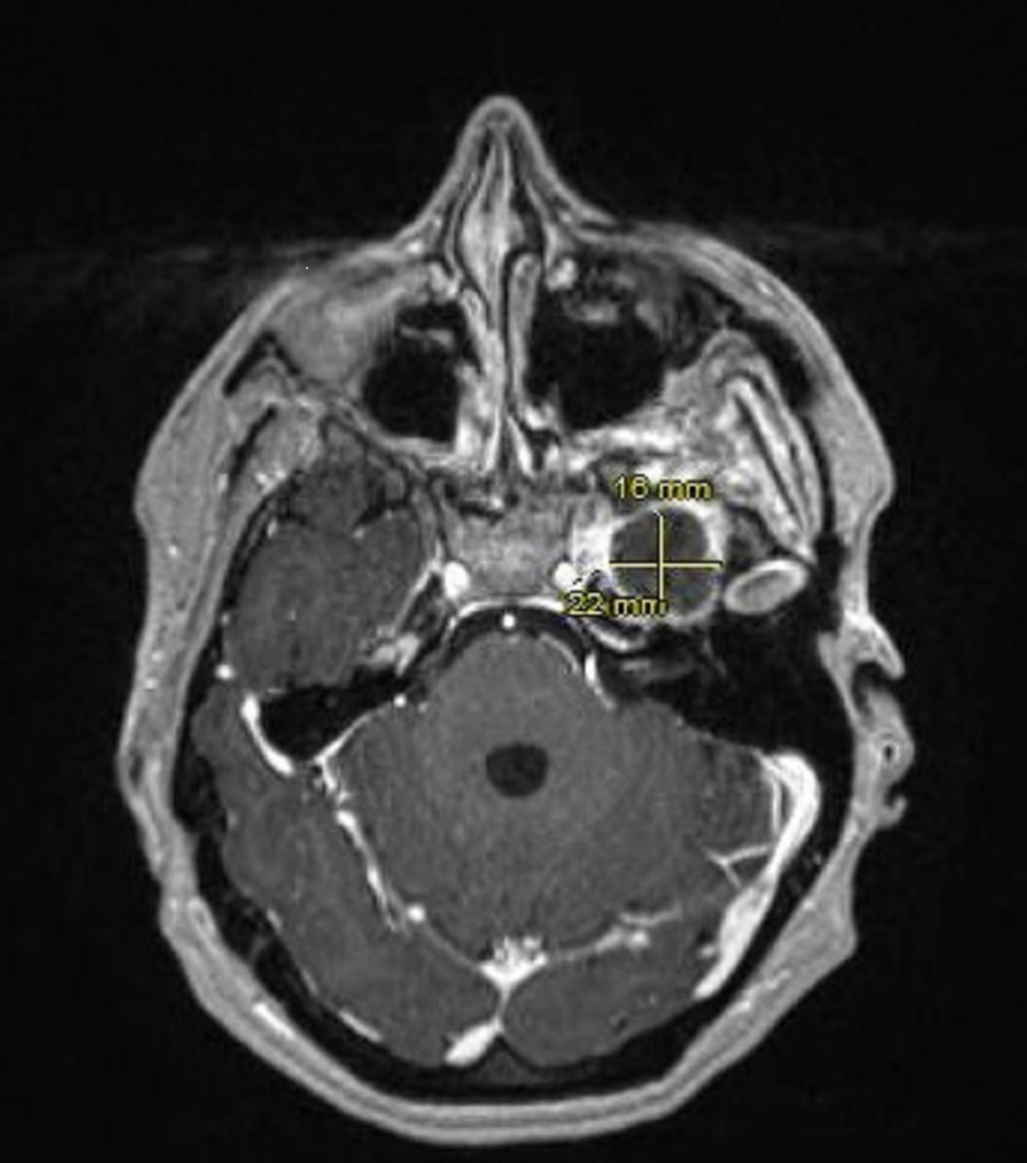
Magnetic resonance imaging (MRI) brain showing 22 x 16 mm soft tissue mass involving left trigeminal ganglion.

**Figure 2:**
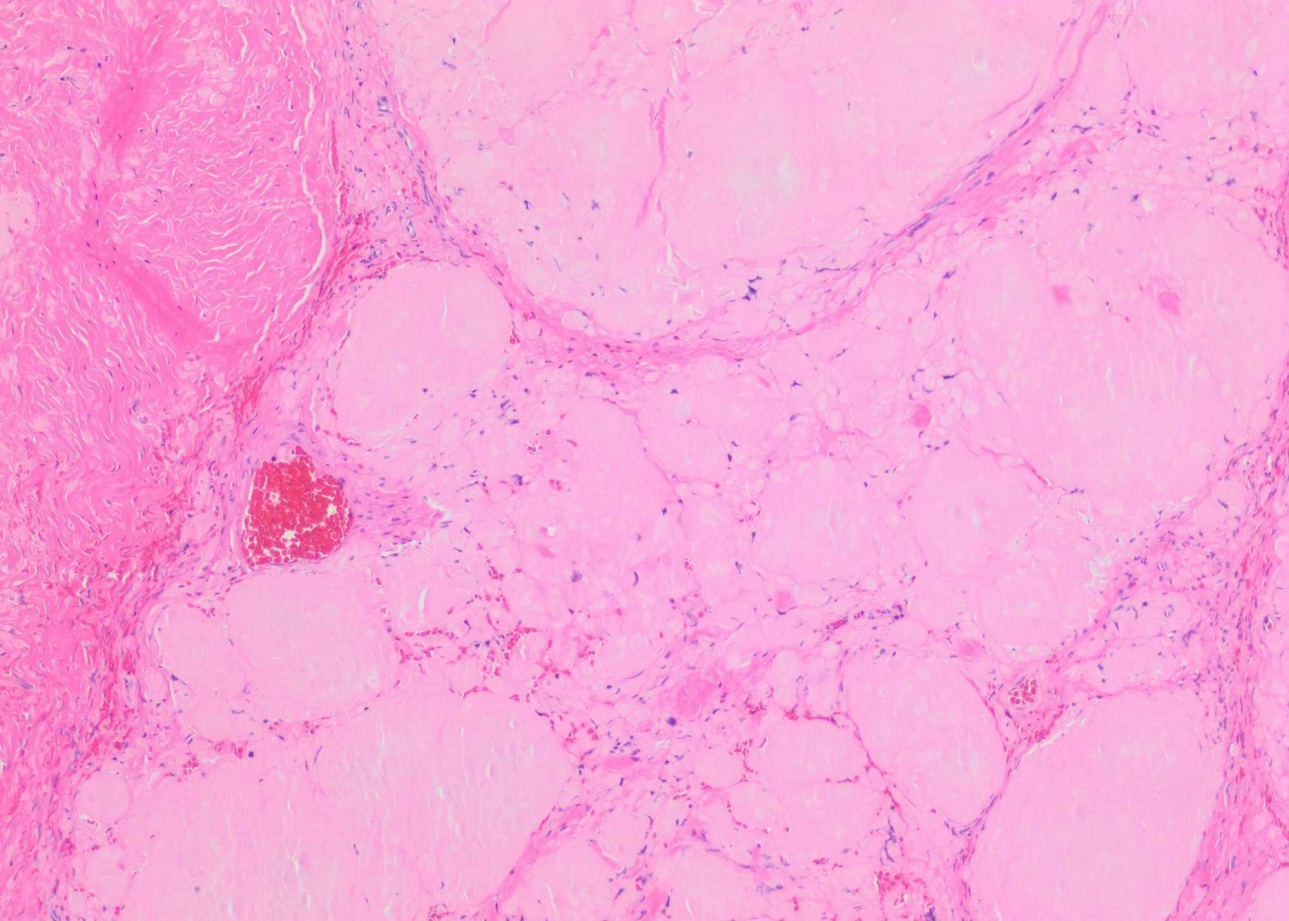
Haematoxylin and eosin (H&E)-stained histologic sections demonstrate abundant amorphous eosinophilic material suspicious for amyloid.

**Figure 3:**
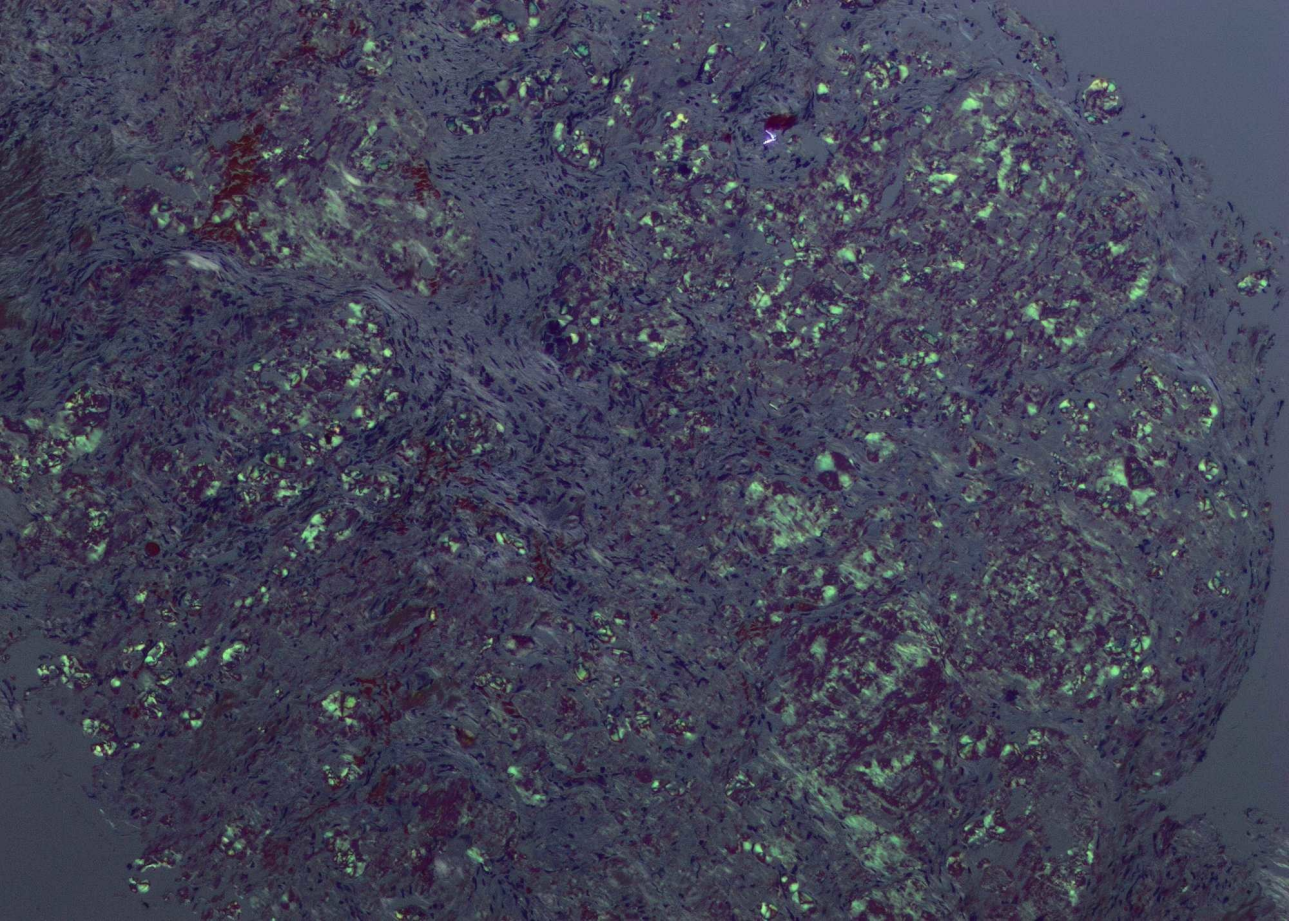
Congo red stain demonstrating "apple-green" birefringence under polarized light, diagnostic of amyloid.

A 34-year-old woman with no significant past medical history presented with the chief complaint of left facial numbness, left ear pain and decreased hearing in the left ear of three years duration. The patient had also been experiencing sharp and shooting pain in different areas of her left thigh. The pain was not associated with any weakness, tingling or numbness. Besides mild fatigue she denied having any fevers, night sweats or weight loss. Neurological physical examination was grossly intact except for sensory loss in the V2 (maxillary) and V3 (mandibular) distribution of trigeminal nerve (cranial nerve V). Abdominal exam was without evidence of lymphadenopathy and hepatosplenomegaly. Given deficits in the sensory distribution of trigeminal nerve, she was initially evaluated by ear, nose and throat (ENT) and underwent two sequential minimally invasive surgeries for nasal polyps without significant resolution of her symptoms. She was subsequently referred to a neurologist and had magnetic resonance imaging (MRI) of the brain performed. Brain MRI revealed a soft tissue mass with expansion in the left Meckel’s cave, measuring 22 x 16 x 12 mm (Figure 1), raising concerns for a trigeminal schwannoma. She was evaluated by the neurosurgery and underwent an orbital zygomatic craniotomy and left trigeminal schwannoma resection. Pathology revealed deposition of abundant hypocellular eosinophilic material on light microscopy examination (Figure 2). Congo red staining demonstrated characteristic 'apple-green birefringence' upon polarization (Figure 3), consistent with diagnosis of trigeminal nerve amyloidoma. Unfortunately, no immune fluorescence or electron microscopy was done on the pathology specimen to determine the amyloid subtype. Postoperatively, the patient was referred to oncology to rule out systemic amyloid deposition. Basic workup including complete blood count was unremarkable except for hemoglobin of 12 g/dL with mean corpuscular volume (MCV) of 76 fL per cell. White blood cell and platelet counts were within normal limits. Comprehensive metabolic panel did not reveal any liver or renal abnormalities. Coagulation profile including prothrombin time (PT), activated partial thromboplastin time (APTT), international normalised ratio (INR) was normal. The patient had both serum and urine protein electrophoresis with immunofixation done which did not reveal any monoclonal protein. Serum free light chains were normal with kappa free light chain 16 mg/dL, lambda free light chain 12 mg/dL and kappa lambda free light chain ratio of 1.36. Urinalysis was without evidence of hematuria or proteinuria. Electrocardiogram (EKG) revealed normal sinus rhythm. Two-dimensional (2D) echocardiogram revealed ejection fraction 60% and normal ventricular wall thickness. Abdominal fat pad biopsy as well as a bone marrow biopsy was performed and both were without evidence of amyloidosis or other plasma cell dyscrasia or lymphoproliferative disorder. The patient was not offered any more localized and systemic therapy with follow-up brain MRI three months after surgical resection without evidence of recurrent amyloidoma.

## Discussion

Introduction

Amyloidosis is characterized by extracellular deposition of an amorphous and heterogeneous protein in body tissues and organs. It can occur as an isolated disease (primary amyloidosis) or as a secondary disease associated with an underlying chronic inflammation, non-inflammatory conditions such as plasmacytomas, chronic renal failure and rheumatoid arthritis [1]. Amyloidoma is defined as primary solitary amyloidosis where no plasma-cell dyscrasia or abnormal serum proteins are detectable [2]. The most common manifestations of central nervous system (CNS) involvement include cerebral amyloid angiopathy and cortical senile plaques associated with Alzheimer disease. Amyloid deposits have also been described along peripheral nerves, in autonomic ganglia and in neurofibrillary tangles in association with dementia [2-6]. Primary CNS amyloidoma is a rare entity with only a few case reports in the literature. Most intracranial amyloidomas occur in cerebral white matter, leptomeninges and spinal cord [7, 8]. Other locations that have been previously reported include Meckel’s cave, gasserian ganglion, skull base, cerebellopontine angle, pituitary gland, temporal, and orbital bone [3, 4, 7, 9]. Isolated trigeminal amyloidomas are rare tumors with only 16 cases described in the literature [3-7, 9-14].

Pathogenesis

The reasons for amyloid deposition in the central nervous system are unclear. Laeng et al. have theorized that amyloidomas are neoplasms of AL (primary) lambda producing B cell clone capable of terminal differentiation in reaction to unique antigens [15]. Cohen et al. suggested that genetically predisposed microglia might be the source of amyloid when challenged by certain antigens [7, 16]. Diagnosis depends on histological demonstration of amyloid in tissue with Congo red staining and subsequent differentiation between AA (secondary) and AL types. In most of the literature cases, the protein subunit of the amyloid was AL lambda [11, 16]. In our case, the amyloid type was determined immunohistochemically as AL lambda. On light microscopic examination, amyloid appears as an eosinophilic amorphous hyaline extracellular substance with characteristic apple green birefringence under polarized light and Congo red staining.

Presentation

Reported cases of Gasserian ganglion amyloidomas predominantly occurred at a mean age of 48 years with slight female predominance [6, 7, 10, 17]. These are slow growing lesions with a mean duration of symptoms of 6.3 years before the diagnosis [4, 7, 10, 11]. In most of these cases, the common presenting symptoms include pain and numbness in one or more trigeminal nerve branches. Amyloidomas can spread perineurally involving motor components and can cause trigeminal neuropathy [6, 7, 10, 11, 13]. The reason for this predilection for trigeminal ganglion is unclear. The other common symptoms associated with intracranial amyloidomas include seizures and cognitive decline [7].

Imaging

Central nervous system amyloidomas typically appear on computed tomography (CT) scan as hypodense patchy lesions with intense enhancement following contrast administration. These lesions have been predominantly described as hyper to hypo intense lesions on T1 weighted MRI and heterogeneous on T2 weighted MRI [17]. Gasserian ganglion amyloidomas appear as brightly enhancing and swollen segments of Cranial nerve V within Meckel’s cave [7, 9, 11].

Prognosis and treatment

The clinical course of trigeminal ganglion amyloidoma is thought to be benign. These lesions grow slowly and symptoms are typically present for years before the diagnosis. The natural history of this rare lesion is poorly known due to low incidence of the disease and few published reports. Overall there have been 16 reports of patients with trigeminal amyloidomas since the first case reported in 1957 (Table 1).

**Table 1:** Summary of all reported cases of trigeminal amyloidoma. GG: Gasserian ganglion; MC: Meckel cave; V1: Ophthalmic; V2: Maxillary; V3: Mandibular.

Patient No.	Reference	Age	Sex	Presentation	Location	Treatment
1	Daly et al., 1957 [5]	42	M	V2 V3 trigeminal pain and numbness	Right GG	Craniotomy with resection
2	Plogsties, 1964 [18]	54	F	V1 V2 V3 numbness and pain	Right GG	Craniotomy for biopsy
3	Borghi and Tagliabue, 1961 [19]	58	F	V1 V2 V3 incomplete numbness, pain and weakness	Right MC	Craniotomy with resection
4	DeCastro et al., 1976 [20]	59	M	V2 V3 numbness and pain progressing to V1	Right GG	Craniotomy with resection
5	Bornemann et al., 1993 [4]	32	F	V2 V3 numbness and pain, atrophy of mastication muscles	Left GG	Craniotomy with resection
6	Bornemann et al., 1993 [4]	49	F	V1 V2 V3 numbness and pain	Left MC	Craniotomy with resection
7	Bornemann et al., 1993 [4]	45	F	V1 V2 V3 numbness	Left MC	Craniotomy with resection
8	O'Brien et al., 1994 [11]	49	F	V1 V2 V3 decreased left sensation, V3 weakness, left V2 pain	Left GG	Craniotomy for biopsy
9	Kirch et al., 1998 [14]	34	M	Bilateral numbness, V1 >V2-V3	Bilateral GG	Craniotomy for biopsy
10	Vorster et al., 1998 [3]	46	F	V2 V3 numbness and pain	Right GG	Craniotomy for resection
11	Matsumoto et al., 1999 [9]	41	F	V2 V3 numbness	Left MC	Craniotomy for resection
12	Yu and de Tilly, 2004 [12]	62	M	Trigeminal pain	Left MC	Craniotomy for biopsy
13	Gottfried et al., 2007 [10]	64	M	V2 V3 numbness	Right MC	Craniotomy for resection
14	Bookland et al., 2007 [7]	32	F	Right facial numbness and pain	Right MC, Foramen Ovale	Craniotomy for resection
15	Yamazaki et al., 2010 [13]	62	F	V1 V2 V3 numbness and weakness	Right MC, V2, V3	Craniotomy for biopsy
16	Gultasli et al., 2012 [6]	57	F	V2-V3 numbness and pain progressing to V1	Bilateral MC	Craniotomy for resection
17	Present Case, 2017	34	F	V2 V3 facial pain and numbness	Left MC	Craniotomy for resection

The reports from all the 16 cases show that authors had similar treatment approaches. Most (11 out of 16) of these patients underwent a craniotomy with surgical resection of the lesion (Table 1). Five patients underwent craniotomy for biopsy of the infiltrated trigeminal ganglion (Table 1). In most of the cases the data is limited on surgical follow-up and long-term imaging was not provided. Thus it is unclear whether the surgical resection and biopsy affected the natural history of the disease. Overall, surgical resection and debulking of tumor outside the capsule has been shown to be effective in alleviating trigeminal pain and dysesthesia. Bornemann et al. reported that two patients with postoperative sensory and motor deficits were followed for one and nine years, without a clinical recurrence of the symptoms [4]. Focused radiotherapy, steroids and colchicine have been reported in few case reports without any positive results [7, 11, 15].

In most of these cases extensive postoperative workup was performed to rule out systemic causes of amyloidosis. Investigative workup that was performed included thorough blood work, serum protein electrophoresis (SPEP), urine protein electrophoresis (UPEP), serum kappa and lambda light chains, urine kappa and lambda light chains, chest X-ray, electrocardiogram, CT scans of the chest and abdomen, X-rays of the skeleton, nuclear bone scan and echocardiography [1]. In a few cases abdominal fat pad biopsy and bone marrow biopsy was performed to rule out primary disease [6, 11].

## Conclusions

This case demonstrates that in addition to meningiomas, schwannomas, malignant cavernous sinus tumors, and inflammatory disorders, amyloidomas should be considered as a possibility in the differential diagnosis of a cavernous sinus lesion or of a lesion arising from the Gasserian ganglion or within Meckel's cave. Once the diagnosis is established, the workup should be done to rule out the presence of systemic amyloidosis including fat pad and/or bone marrow biopsy. In the absence of systemic involvement, localized amyloidosis in the form of trigeminal amyloidoma can be effectively treated with surgical resection with relief of symptoms.
